# Characterizing Genetic Diversity of Contemporary Pacific Chickens Using Mitochondrial DNA Analyses

**DOI:** 10.1371/journal.pone.0016843

**Published:** 2011-02-04

**Authors:** Kelsey Needham Dancause, Miguel G. Vilar, Rlene Steffy, J. Koji Lum

**Affiliations:** 1 McGill University/Douglas Hospital Research Center, Montreal, Canada; 2 Department of Anthropology, University of Pennsylvania, Philadelphia, Pennsylvania, United States of America; 3 Micronesian Area Research Center, University of Guam, Hagåtña, Guam; 4 Department of Anthropology, Binghamton University, Binghamton, New York, United States of America; University of Utah, United States of America

## Abstract

**Background:**

Mitochondrial DNA (mtDNA) hypervariable region (HVR) sequences of prehistoric Polynesian chicken samples reflect dispersal of two haplogroups—D and E—by the settlers of the Pacific. The distribution of these chicken haplogroups has been used as an indicator of human movement. Recent analyses suggested similarities between prehistoric Pacific and South American chicken samples, perhaps reflecting prehistoric Polynesian introduction of the chicken into South America. These analyses have been heavily debated. The current distribution of the D and E lineages among contemporary chicken populations in the Western Pacific is unclear, but might ultimately help to inform debates about the movements of humans that carried them.

**Objectives:**

We sought to characterize contemporary mtDNA diversity among chickens in two of the earliest settled archipelagoes of Remote Oceania, the Marianas and Vanuatu.

**Methods:**

We generated HVR sequences for 43 chickens from four islands in Vanuatu, and for 5 chickens from Guam in the Marianas.

**Results:**

Forty samples from Vanuatu and three from Guam were assigned to haplogroup D, supporting this as a Pacific chicken haplogroup that persists in the Western Pacific. Two haplogroup E lineages were observed in Guam and two in Vanuatu. Of the E lineages in Vanuatu, one was identical to prehistoric Vanuatu and Polynesian samples and the other differed by one polymorphism. Contrary to our expectations, we observed few globally distributed domesticate lineages not associated with Pacific chicken dispersal. This might suggest less European introgression of chickens into Vanuatu than expected. If so, the E lineages might represent lineages maintained from ancient Pacific chicken introductions. The Vanuatu sample might thus provide an opportunity to distinguish between maintained ancestral Pacific chicken lineages and replacement by global domesticates through genomic analyses, which could resolve questions of contemporary haplogroup E chicken relationships and inform interpretations of debated sequences from archaeological samples.

## Introduction

The discovery and settlement of the thousands of islands of Micronesia, Polynesia, and Eastern Melanesia beginning around 3,500 years ago [1] and continuing to as recently as 800 years ago [2] presented countless difficulties, including the transport of domestic animals and their maintenance on islands of greatly varying sizes and environmental resources. The Lapita Cultural Complex, the package of material culture associated with the settlers of Eastern Melanesia and Polynesia, included introduced Island Southeast Asian and indigenous Melanesian domesticates that were central in overcoming these constraints. Three of these domesticates – the Pacific pig, chicken, and dog – along with the commensal Polynesian rat, provided important food sources in the tropics and, notably, could be carried in outrigger canoes and were thus transported widely across the Pacific [1], [3].

The distribution of these domesticates can inform our understandings of human movements [4]–[7], and the distribution of chickens has gained a great deal of recent attention. In 2007, Storey and colleagues [8] analyzed mitochondrial DNA (mtDNA) hypervariable region (HVR) sequences among prehistoric Polynesian chicken samples and, based on similarities between these and lineages from both ancient and contemporary chicken samples in Chile, suggested that pre-Columbian Pacific voyagers introduced chickens into South America [8], [9]. These conclusions were questioned by Gongora and colleagues [10], [11], who suggested that correcting the radiocarbon dates of the ancient Chilean samples pushes their age to a post-Columbian period, and the similarities between both the ancient and contemporary Chilean lineages and contemporary European domesticates supports European introduction of chickens into South America. Storey et al. responded with analyses of ancient chicken samples from Vanuatu [12], the first archipelago settled by Lapita colonizers around 3000 years ago [1]. These samples were identical to other ancient Polynesian samples, further supporting prehistoric Pacific introduction of this lineage into the Pacific, but not clarifying the issue surrounding the origins of the contemporary Chilean chickens that Gongora et al. [10], [11] questioned.

The issue remains unresolved because the data currently available are consistent with both prehistoric and historic introductions. Storey and colleagues [8], [9] observed two prehistoric Pacific haplogroups. The most geographically widespread falls into haplogroup E as defined by Liu et al. [13]. Samples dating from 3200/3000 B.P. to 500 B.P. in Vanuatu, Tonga, American Samoa, Niue, Hawai'i, and Easter Island were identified as haplogroup E, also observed among the ancient Chilean samples, and among contemporary Araucana Chilean chickens. Coincidently, the two haplogroup E lineages identified (E1 and E6) are also distributed globally today among chickens from China, Japan, India, the Middle East, Europe, and South America [10]. They are also found at particularly high frequencies among common commercial breeds. The second prehistoric Pacific lineage is associated with haplogroup D [or haplogroup C as defined by Oka et al. [14]; see also [10]]. Five out of six Easter Island samples dating from about 1210–1430 A.D. [8] fall into this haplogroup, which also includes contemporary samples from Indonesia, the Philippines, Thailand, Myanmar, Madagascar, India, Okinawa, and southern China [10].

**Figure 1:**
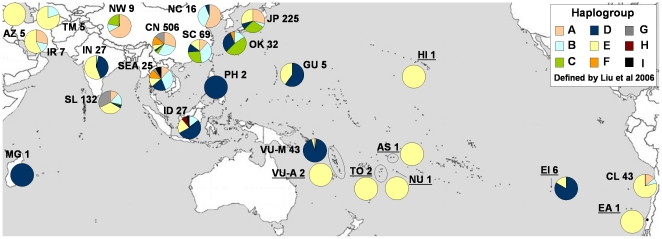
Frequency of chicken haplogroups by geographic location: sampling location followed by sample size. Reference, Accession Numbers: AS: American Samoa (8, EF535240); AZ: Azerbaijan (13, AY704696-700); CL: Chile (10, EF190830-70); CN: Central China – Guizhou, Guangxi, Henan, Hubei, Hunan, Jiangxi, Sichuan, and Yunnan provinces (13, AF512057-060, 062-179, 189-214, 234-254, 261-337, AY392172-407; 15, AB098664-6; 16, AY465968-5975, 5988-6003; 17, AY588613-20, 29-35); EA: El-Arenal 1 (8, EF535241); EI: Easter Island (8, EF535242-7); GU: Guam (this study, FJ914362-6); HI: Hawaii (8, EF535238); ID: Indonesia (13, AY642127-33; 18, D82916-9; 19, AB007726, AB009436-40; 20, AB268525-28, 40, 45); IN: India (13, AY644966-73, AY704701-19); IR: Iran (13, AY704720-4; 19, AB009444; 21, FJ619040); JP: Japan (15, AB098636-39, 41-55, 57-63, 70-87, 89-95, 97-99; 18, D82921, 3, 5; 19, AB007722, 28-33, 35-41, 43-48, 50-51, 54-55, 58, AB009427-29, 46; 20, AB268506-24, 29-37, 39; 22, AB114058-86); MG: Madagascar (19, AB007742); NC: North Coastal China – Shandong and Liaoning provinces (16, AY465976-79, 84-87; 17, AY588621-8); NU: Niue (8, EF535239); NW: Northwest China – Xinjiang and Tibetan provinces (16, AY465960-5963; 17, AY588608-12); OK: Okinawa (15, AB098638-39, 46, 51-55, 57-63, 92-95, 97-99; 20, AB268538, 41-44); PH: Philippines (19, AB009433; 23, AP003322); SC: South Coastal China – Fujian, Guangdong, Jiangsu, and Zhejiang provinces (13, AF512215-33, 55-60; 16, AY465964-5966, 5980-5983, 6004-6007; 17, AY588636-42; 19, AB007734, 49; 24, AF128315-44); SL: Sri Lanka (25, EU199906-47); SEA (Southeast Asia): Laos (19, AB009442, 8; 23, AP003319), Malaysia (13, AY642134), Myanmar (13, AF512180-8; 15, AB098667-9), Thailand (19, AB007724, AB009432, 41, 43; this study, FJ914360-1), Vietnam (19, AB009434-35, 49); TM: Turkmenistan (13, AY704725-9); TO: Tonga (8, EF535236-7); VU-A: Vanuatu-Ancient (12, HM189678-9); VU-M: Vanuatu-Modern (this study, FJ914317-59).

The distribution of haplogroup D in Southeast Asia, Island Southeast Asia, and Polynesia, and its scarcity elsewhere (Figure 1), suggests that this indeed represents a “Pacific chicken” lineage, and that the ancient Easter Island samples analyzed by Storey et al. [8] reflect a part of the original dispersal of chickens into the Pacific. However, the degree to which this lineage has been maintained in the Western Pacific is unclear [10]. Furthermore, whereas data from Storey et al. [8], [12] indicate that lineages E1 and E6 were transported prehistorically throughout the Pacific, we have little data on their contemporary distribution in Remote Oceania.

We sought to characterize genetic diversity of contemporary chickens in Vanuatu and, in particular, to examine whether the D and E lineages are present today. We also included analyses of contemporary samples from Guam in the Mariana archipelago, another of the earliest settled regions of Remote Oceania. We collected samples from contemporary chicken populations in these two regions (Vanuatu n  =  43, Guam n  =  5), analyzed mtDNA HVR sequences, and compared them to sequences from contemporary and prehistoric samples from the Asia-Pacific region. We expected to observe diversity among contemporary chicken populations, but hypothesized that lineages observed among ancient Pacific samples would be maintained in Remote Oceanic populations. These analyses not only help to highlight the current distribution of chicken lineages in the Pacific, but also provide a framework for discussing the complications inherent in distinguishing prehistoric, historic, and recent commercial introductions and in inferring origins based on current lineage frequencies and distributions.

## Materials and Methods

Samples were collected and analyzed in accordance with Binghamton University Division of Research guidelines. Following the Binghamton University Institutional Review Board (IRB) guidelines, samples were taken with animal owners' consent and without harm to the animals involved; full IRB review was not necessary. Chicken feathers were collected from 12 sampling sites representing nine villages and four islands in the Vanuatu archipelago: Ambae, Aneityum, Efate, and Tanna (see Figure S1), and from Guam in the Mariana Islands. Most feathers were plucked from live chickens by their owners or the researchers. Some were collected from roosts or butchering sites; only one feather was taken from each of these sites to avoid duplicate sampling. Although the pedigrees of the birds cannot be confidently ascertained, multiple feathers collected from the same neighborhood/sampling site were taken from different households to avoid sampling sibling or parent-offspring pairs.

The cumulus of feathers was washed with ethanol, rinsed with distilled water, and cut into small pieces. DNA was extracted using the QIAamp DNA Tissue Kit (Qiagen Biosciences, Germantown, MD) according to the manufacturer's instructions, with the addition of preliminary overnight incubation in Proteinase K at 56 degrees. DNA was amplified by PCR using primers 5′-ACCCATTATATGTATACGGGCATTAA-3′ and 5′-AGTTATGCATGGGATGTGCCTGACCGAG-3′. Approximately 420 bases of the mtDNA control region were sequenced in both directions with the BigDye Terminator Kit on an ABI 3730xl DNA analyzer (Applied Biosystems, Foster City, CA).

Phylogenetic analysis: 420 base pairs from the mtDNA HVR (accession numbers FJ914317-59, FJ914362-6) were aligned and compared to published sequences [8], [10], [13], [15]–[24] (accession numbers are listed in the legend for Figure 1). Haplogroups were assigned following the nomenclature of Liu et al. [13]. A map and table illustrating the frequency of chicken haplogroups, standardized to the nomenclature of Liu et al. [13], was generated from submitted sequences and published accounts to illustrate patterns of Asian and Oceanic chicken diversity (Figure 1).

## Results

Twenty-four polymorphic sites were identified across 420 bp of mtDNA HVR from comparisons of the Vanuatu and Guamanian samples to the reference sequence [13] (see Table 1; table includes 25 polymorphic sites, one of which was specific to an ancient Vanuatu sample analyzed by Storey et al. [12]). Three of the five Guamanian samples and 40 of the 43 Vanuatu samples were assigned to haplogroup D. This haplogroup was found on all four islands sampled in Vanuatu. Four haplogroup E samples were observed: two in Guam (haplotypes E1 and E3) and two in Vanuatu (haplotype E6 and one differing from haplotype E1 at one single nucleotide polymorphism, SNP). Finally, one sample from Vanuatu was assigned to haplogroup A1. The similarities between samples from different islands in Vanuatu can be observed in Table S1, which lists the polymorphic sites specific to the Vanuatu sequences and clusters related sequences together.

**Table 1:** Nucleotide polymorphisms in ancient and contemporary Remote Oceanic chicken samples.

Type	Locations	HG	n	167	177	199	210	217	225	232	233	238	243	256	261	265	281	293	296	302	306	309	310	324	330	342	363	446
				C	A	T	T	T	T	C	C	G	T	T	C	C	A	T	C	C	T	T	C	A	C	A	C	C
M	Vanuatu (Efate)	A1	1	.	.	.	C	.	.	.	.	.	.	.	.	.	.	.	.	.	.	.	.	.	.	.	.	.
M	Vanuatu (Efate)	E	1	T	.	.	C	C	C	.	.	A	C	C	T	.	.	.	.	.	.	.	T	.	.	.	.	T
A	Chile, Tonga	E1	2	T	.	.	C	C	C	.	.	.	C	C	T	.	.	.	.	.	.	.	T	.	.	.	.	–
A	Am. Samoa	E1	2	–	.	.	C	C	C	.	.	.	C	C	T	.	.	.	.	.	.	.	T	.	.	.	.	–
A	Easter Island	E1	1	–	.	Y	C	C	C	.	.	.	C	C	T	.	.	.	.	.	.	.	T	.	.	.	.	–
M	Guam	E1	1	T	.	.	C	C	C	.	.	.	C	C	T	.	.	.	.	.	.	.	T	.	.	.	.	T
M	Guam	E3	1	T	.	.	C	C	C	.	.	.	C	C	T	.	.	.	.	.	.	.	T	.	T	.	.	T
M	Vanuatu (Efate)	E6	1	–	–	C	C	C	C	.	.	.	C	C	T	.	.	.	.	.	.	.	T	.	.	.	.	T
A	Vanuatu (Teouma Site)	E6	1	–	–	C	C	C	C	.	.	.	C	C	T	.	.	.	.	.	.	.	T	T	.	.	.	.
A	Vanuatu (Teouma Site)	E6/E7	1	–	–	C	C	C	C	.	.	.	C	C	T	.	.	.	.	.	.	.	T	.	.	.	.	.
A	Hawaii, Niue, Tonga	E6	3	T	.	C	C	C	C	.	.	.	C	C	T	.	.	.	.	.	.	.	T	.	.	.	.	T
M	Vanuatu (Ambae, Aneityum, Efate, Tanna)	D6	17	T	.	.	C	.	C	.	.	.	C	C	T	.	G	.	T	.	C	.	T	.	.	G	.	.
A	Easter Island	D6	3	–	.	.	C	.	C	.	.	.	C	C	T	.	G	.	T	.	C	.	T	.	.	G	.	–
A	Easter Island	D6	2	T	.	.	C	.	C	.	.	.	C	C	T	.	G	.	T	.	C	.	T	.	.	G	.	–
																												
M	Vanuatu (Tanna)	D1	1	T	T	.	C	.	C	.	.	.	C	C	T	T	G	.	.	.	C	.	T	.	.	G	T	.
M	Guam	D	1	T	.	.	C	.	C	.	.	.	C	C	T	T	G	.	T	.	C	.	.	.	.	G	.	.
M	Vanuatu (Aneityum)	D	1	T	.	.	C	.	C	T	.	.	C	C	T	.	G	.	T	.	C	.	T	.	.	.	.	.
M	Vanuatu (Tanna)	D	1	T	.	.	C	.	C	.	T	.	C	C	T	.	G	.	T	.	C	.	T	.	.	G	.	.
M	Vanuatu (Ambae, Aneityum, Efate)	D	5	T	.	.	C	.	C	.	.	.	C	C	T	.	G	.	T	.	C	.	T	.	.	.	.	.
M	Guam	D13	1	T	.	.	C	.	C	.	.	.	C	C	T	.	G	.	.	.	C	.	T	.	.	.	.	.
M	Guam	D	1	T	.	.	C	.	C	.	.	.	C	C	T	.	G	.	.	T	C	C	T	.	.	.	.	.
M	Vanuatu (Ambae, Efate)	D	4	T	.	.	C	.	C	.	.	.	C	C	T	.	G	.	.	.	C	C	T	.	.	.	.	.
M	Vanuatu (Aneityum)	D	2	T	.	.	C	.	C	.	.	.	C	C	T	.	.	C	T	.	C	.	T	.	.	G	.	.
M	Vanuatu (Aneityum)	D	9	T	.	.	C	.	C	.	.	.	C	C	T	.	.	.	T	.	C	.	T	.	.	G	.	.

HG  =  haplogroup, n  =  sample size. M  =  Modern, A  =  Ancient.

– indicates sequence unavailable. Note: In contrast to the numbering of Storey et al. [8], [12], base positions in this table are numbered to match Liu et al. [13]. To compare them to Storey et al. [8], [12] each position must have three subtracted from it (i.e. 210 in this table is the same as 207 in Storey et al).

Among the three Guamanian haplogroup D samples, three lineages were observed. One matched contemporary samples from the Philippines, Indonesia, and Japan. The other lineages were unique, but were closely related to contemporary samples from Indonesia, Japan, the Philippines, and China as well as India, Sri Lanka, Thailand, and Madagascar. Among the 40 haplogroup D samples from Vanuatu, seven lineages were observed. Four were exact matches to contemporary samples from Indonesia, Japan, the Philippines, and Southern China, as well as prehistoric Easter Island samples. The major Vanuatu lineage (n  =  17), also found in a Red Jungle Fowl from the Philippines, was distributed across all four islands sampled (see Table 2).

**Table 2:** Frequencies of contemporary Haplogroup D lineages in Vanuatu and closely related lineages from the Asia-Pacific Region

		Vanuatu				Pacific		SEA			Asia		
		Ambae	Aneytium	Efate	Tanna	Ancient Easter Island	Guam	Indonesia*	Philippines*	Thailand	India*	China	Japan
np	HT	n = 7	n = 19	n = 14	n = 3	n = 6	n = 5	n = 27	n = 2	n = 1	n = 27	n = 600	n = 225
	D6							0.08			0.04		0.03
342	D13						0.20	0.04	0.50	1.00		<0.01	0.03
296		0.57	0.32	0.43	0.33	0.67			0.50				
296, 342		0.29	0.05	0.14									<0.01
281, 296			0.47										
281, 293, 296			0.11										
232, 296, 342			0.05										
296, 342, 391								0.08					
233, 296					0.33								
296, 331t						0.17							
309								0.04					
309, 342		0.14		0.21				0.08					
221, 309, 342	D14							0.04					
302, 309, 342							0.20						
363											0.11		
177t, 265, 363					0.33							<0.01	
265, 310							0.20						

np: nucleotide polymorphisms as compared to haplotype D6 (Liu et al. 2006).

HT: Haplotypes as assigned by Liu et al. [13].

*Samples include both domestic chickens and Red Junglefowl.

## Discussion

Ancient DNA analyses suggest that both D and E lineages were transported into Remote Oceania prehistorically, but there has been little sampling among contemporary populations in Remote Oceania to address the question of whether these patterns remain. Our data indicate that lineages observed among prehistoric Pacific samples are present in Remote Oceania today. The signature of haplogroup D, previously suggested to be erased across the Western Pacific with the introgression of European chickens [10], remains strong in both Guam and Vanuatu.

The lineages observed in Vanuatu were closely related to other ancient Vanuatu and Polynesian samples: the major lineage (n  =  17) matched four of the six prehistoric samples from Easter Island assigned to haplogroup D6, and Vanuatu haplogroup D lineages were also closely related to contemporary samples from southern China, Indonesia, and the Philippines, consistent with the theorized homeland of the Lapita settlers of Remote Oceania [1]. We also observed E lineages in Vanuatu, although at much lower frequency. The small sizes and relative isolation of the sampled islands could contribute to genetic drift, which has likely impacted the frequencies of the lineages observed. The E6 lineage in Vanuatu was identical to prehistoric samples from Vanuatu, Hawai'i, Niue, and Ha'ateiho Tonga, and the other E lineage differed from prehistoric samples from American Samoa, Chile, Easter Island, and Mele Havea Tonga (E1) [8] by one SNP (np 238). Contrary to our expectations, we observed only one globally distributed lineage not associated with Pacific chicken dispersals – haplotype A1. This was notable, considering that reports from 1891 suggested that European chickens had replaced native chicken stocks in Vanuatu [12]; certainly there were numerous opportunities for European chicken introductions by explorers, sandalwood traders, and plantation owners during the 18^th^ and 19^th^ centuries [26]. Nevertheless, we observed Pacific chicken lineages at high frequencies on all islands sampled.

As among the Araucana chickens in Chile, the lineages in our Remote Pacific samples – especially the E lineages, which are observed among contemporary samples around the globe – might have persisted from those introduced prehistorically, they might have been historically introduced, they might reflect recent expansion of commercial domesticates, or they might result from any combination of these patterns. In Guam, for example, one field report documents bird bones up to 2000 years old that may be from chickens [27], but these archaeological findings remain debated and chickens were most likely introduced to the island only historically – possibly from the Philippines, considering the history of the Marianas [28]. Furthermore, globally distributed domesticate lineages common in the United States might have been introduced in recent decades with the growth of the poultry industry. The same is true of Vanuatu. However, this contemporary sample has several characteristics that might make it interesting for future analyses of chicken dispersals. The presence of chickens at securely prehistoric archaeological sites suggests the early transport of Pacific chickens into Vanuatu [12], which might have reduced the motivation for later adoption of European lineages on some islands. Furthermore, the high frequency of haplogroup D in Vanuatu (93% of our sample) and the distribution of the D lineages across all four sampled islands support the maintenance of Pacific chickens in the archipelago. Finally, the absence in our sample of the most common European and global commercial lineages and the presence of only one globally distributed sample not associated with Pacific chicken dispersals might suggest that European domestic chickens have had less impact in Vanuatu than expected.

The maintenance of Pacific chicken lineages in Vanuatu might provide an opportunity to resolve questions about South American chicken origins that have remained unanswered with available data sets. Just as the origins of contemporary haplogroup E lineages in Vanuatu are not firmly known, whether the haplotypes observed among contemporary Chilean Aracauna chickens reflect the persistence of ancient Polynesian haplotypes, the spread of European chickens introduced post-colonially, or the introgression of common commercial lineages today, has not been and cannot be resolved with mtDNA HVR sequences alone. The presence of both Pacific chicken lineages among contemporary Vanuatu samples might suggest that this population contains descendents of the original Pacific chickens. Genomic data from this sample could be compared to data from European and contemporary Chilean haplogroup E lineages, which have otherwise identical mtDNA HVR sequences, to resolve their relationships. While interpretations of past introductions based on contemporary distributions are difficult, the generation of more extensive data sets from carefully selected populations that have maintained Pacific chicken diversity could provide the context necessary for resolving the relationships between contemporary samples, and the interpretations of continuous human movement of domestic animals they inform.

## Supporting Information

Figure S1 Location of Vanuatu in relation to Australia and New Guinea (inset) and sampling locations.
(TIF)

Table S1 Nucleotide polymorphisms in contemporary Vanuatu samples.
(XLS)
