# Supplementary figures and images for: Characterizing Genetic Diversity of Contemporary Pacific Chickens Using Mitochondrial DNA Analyses
Source: PLoS One. 2011 Feb 4;6(2):e16843. doi: 10.1371/journal.pone.0016843 (PMC3033910; doi:10.1371/journal.pone.0016843)

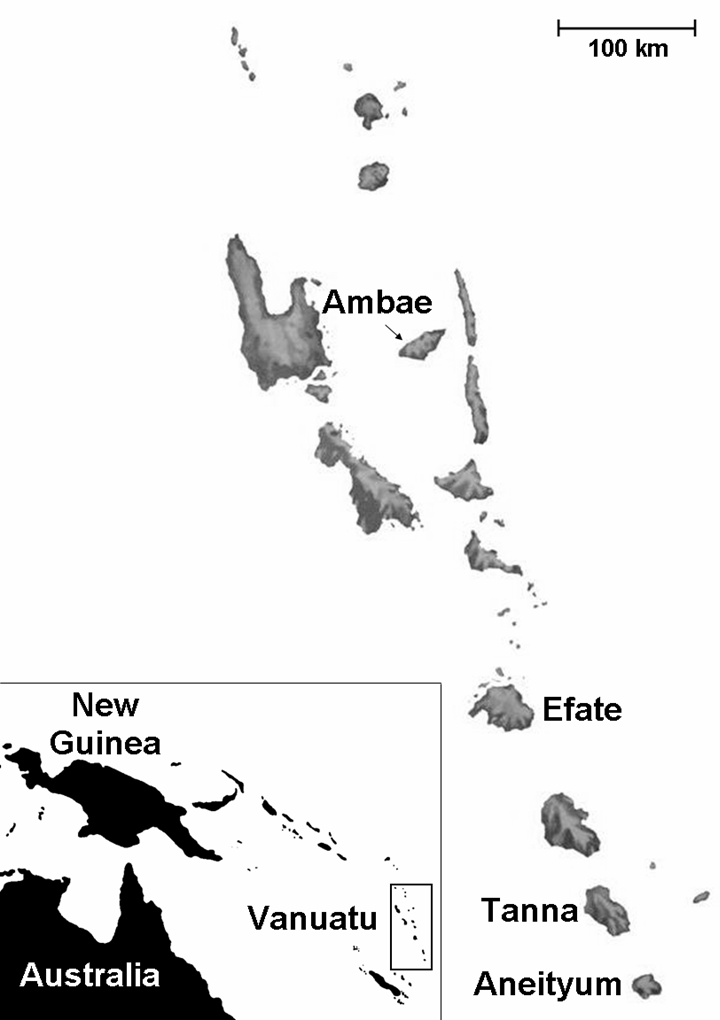

Supplement: Figure S1 — Location of Vanuatu in relation to Australia and New Guinea (inset) and sampling locations. (TIF) [file pone.0016843.s001.tif]
